# Cyclophilins and Their Roles in Hepatitis C Virus and Flavivirus Infections: Perspectives for Novel Antiviral Approaches

**DOI:** 10.3390/pathogens10070902

**Published:** 2021-07-17

**Authors:** Carla E. Gallardo-Flores, Che C. Colpitts

**Affiliations:** Department of Biomedical and Molecular Sciences, Queen’s University, Kingston, ON K7L 3N6, Canada; c.gallardoflores@queensu.ca

**Keywords:** cyclophilins, hepatitis C virus, flaviviruses, virus-host interactions, antiviral therapy

## Abstract

Cyclophilins are cellular peptidyl-prolyl isomerases that play an important role in viral infections, with demonstrated roles in the replication of hepatitis C virus (HCV) and other viruses in the *Flaviviridae* family, such as dengue virus (DENV) and yellow fever virus (YFV). Here, we discuss the roles of cyclophilins in HCV infection and provide a comprehensive overview of the mechanisms underlying the requirement for cyclophilins during HCV replication. Notably, cyclophilin inhibitor therapy has been demonstrated to be effective in reducing HCV replication in chronically infected patients. While the roles of cyclophilins are relatively well-understood for HCV infection, cyclophilins are more recently emerging as host factors for flavivirus infection as well, providing potential new therapeutic avenues for these viral infections which currently lack antiviral therapies. However, further studies are required to elucidate the roles of cyclophilins in flavivirus replication. Here, we review the current knowledge of the role of cyclophilins in HCV infection to provide a conceptual framework to understand how cyclophilins may contribute to other viral infections, such as DENV and YFV. Improved understanding of the roles of cyclophilins in viral infection may open perspectives for the development of cyclophilin inhibitors as effective antiviral therapeutics for HCV and related viruses.

## 1. Introduction

Approximately 71 million people around the world are chronically infected with hepatitis C virus (HCV) [1]. Chronic HCV infection significantly increases the risk of progressive liver disease leading to cirrhosis and hepatocellular carcinoma, and is one of the leading causes of liver transplantation in the U.S. [2,3]. As of 2015, the most affected World Health Organization (WHO) regions were the Eastern Mediterranean Region and the European Region, with HCV prevalence estimates of 2.3% and 1.5%, respectively [4]. HCV is genetically diverse and classified into six major genotypes with distinct geographical distributions [5]. Genotypes 1 and 3 are the most prevalent worldwide (46.2% and 30.1% of cases, respectively), whereas genotypes 4 and 5 are more common in lower-income countries [5]. HCV is spread mainly through the blood and, despite intensive efforts, there is still no effective vaccine to prevent HCV infection. Despite the remarkable development of effective direct-acting antivirals (DAAs) allowing for the cure of chronic HCV infection in the majority of patients, DAA therapy remains prohibitively expensive and is not accessible for all patients. For example, the extended treatment duration for patients with compensated cirrhosis becomes unaffordable at current DAA prices [6]. Furthermore, emergence of resistance to DAAs can lead to treatment failure [7,8,9], and rarer genotypes (such as those more common in lower-income countries) are more difficult to treat [5]. Investigation into alternative therapeutic approaches is still warranted.

HCV, classified in the *hepacivirus* genus of the *Flaviviridae* family, is an enveloped virus with a positive-sense single-stranded RNA ((+)ssRNA) genome [10]. The HCV genome is approximately 9 kb in length, and encodes a polyprotein that is processed into three structural (core, E1 and E2) and seven non-structural proteins (p7, NS2, NS3, NS4A, NS5A and NS5B). The viral replication cycle is initiated by the interaction of the E1 and E2 glycoproteins with several receptors on hepatocytes [11]. Following a complex post-binding entry process, HCV is internalized via clathrin-mediated endocytosis and undergoes membrane fusion dependent on low pH within the endosomal compartment [11]. Upon uncoating and release of the (+)ssRNA genome in the cytoplasm, the viral RNA is directly translated by the cellular ribosomal machinery into a single polyprotein precursor of ~3000 amino acids. This polyprotein is cleaved by NS2/3 and NS3/4A viral proteases [12] and by host signal peptide protease [13,14] into the structural and non-structural proteins.

HCV non-structural proteins NS4B and NS5A drive the remodeling of intracellular membranes into a “membranous web” comprising the replication organelle (RO) [15]. The RO serves as a platform for viral RNA replication and also shields the replicating viral RNA from innate immune sensors [16]. Within the RO, the (+)ssRNA genome serves as the template for the viral RNA-dependent RNA polymerase (RdRp) NS5B to generate negative-sense RNA intermediates that are used as templates to generate more positive-sense genomes [17]. Viral assembly is thought to be regulated by phosphorylation of NS5A [18,19], culminating in the delivery of (+)ssRNA genomes to core protein on cellular lipid droplets (LDs), where the process of viral assembly occurs, forming the nucleocapsid. The virion envelope is acquired by the budding of the nucleocapsid into the ER at sites of lipoprotein synthesis [20]. HCV particles egress through the lipid secretory pathway and become associated with lipoproteins to produce lipoviroparticles (LVPs) [21].

**Figure 1:**
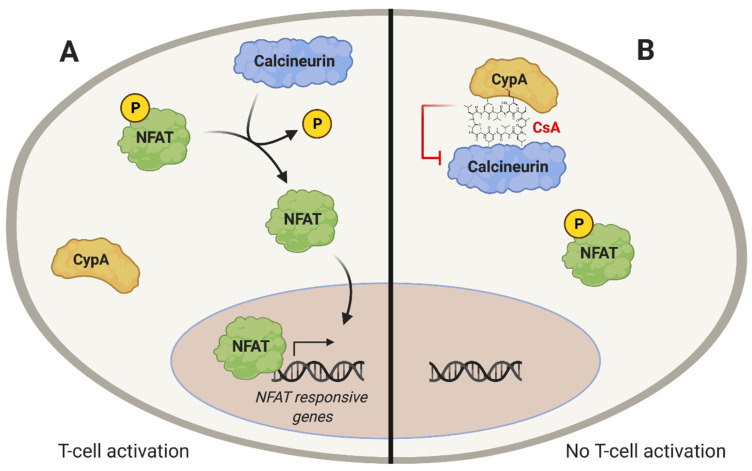
Immunosuppressive effect of CsA. (**A**) T-cell activation depends on the dephosphorylation of the transcription factor nuclear factor of activated T-cells (NFAT). Calcineurin catalyzes dephosphorylation of NFAT, enabling its nuclear translocation. (**B**) The CypA inhibitor, CsA, binds to CypA, enabling the CypA-CsA complex to bind to and inhibit calcineurin, thus preventing dephosphorylation and nuclear translocation of NFAT.

HCV replication, like that of other viruses, relies on interactions with host factors. Cyclophilins (Cyps) are a family of cellular proteins that have roles in the replication of many viruses. The first cyclophilin that was identified, cyclophilin A (CypA), was described as a cytosolic protein that could bind to the immunosuppressive drug cyclosporin A (CsA) in lymphoid cells [22]. The discovery of CypA as the target of CsA allowed the understanding of the immunosuppressive effects of CsA, which had been of long-standing interest due to the potential uses of CsA in organ transplantation. However, the molecular mechanisms underlying immunosuppression by CsA were not understood until Handschumacher et al. identified CypA as a CsA target [22]. CsA-mediated immunosuppression results from the binding of the CypA-CsA complex to calcineurin (Figure 1A), which inhibits the calcineurin-dependent dephosphorylation of the nuclear factor of activated T-cells (NFAT), a transcription factor required for T-cell activation (Figure 1B) [23,24,25]. Importantly, non-immunosuppressive CsA derivatives and other CypI have been developed that retain Cyp-binding, but do not interact with calcineurin, thus allowing for Cyp-inhibitory activity in the absence of immunosuppression [26,27,28,29,30,31,32]. These non-immunosuppressive cyclophilin inhibitors (CypI) may be useful as antiviral molecules.

**Figure 2:**
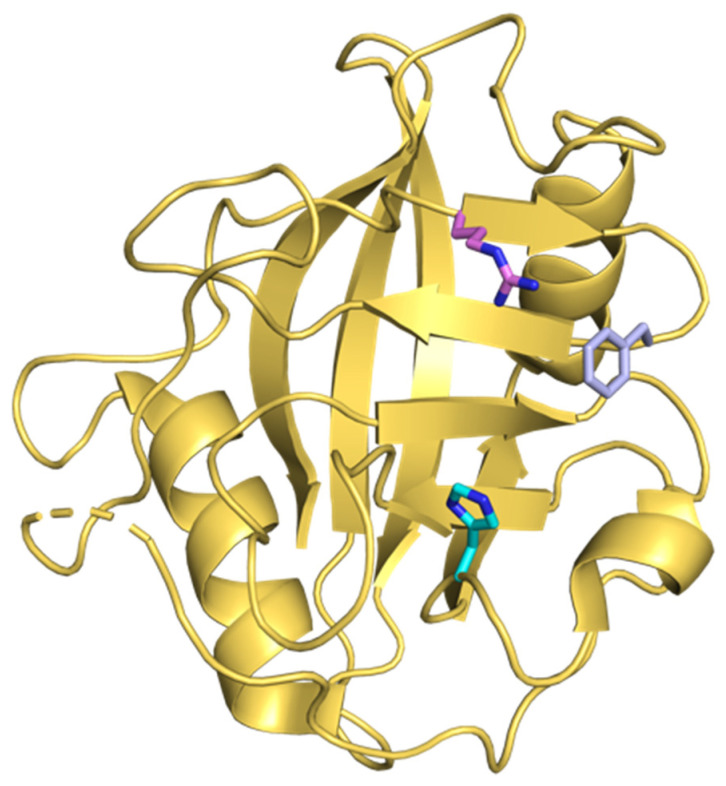
Crystal structure of CypA. PPI active site residues R55 (pink), F60 (violet) and H126 (cyan) are shown. The structure was rendered in PyMOL (PDB: 4IPZ).

CypA, like other Cyps, has peptidyl-prolyl isomerase (PPIase) activity, and Cyps are thought to aid the folding and assembly of other proteins [33]. Cyps catalyze the isomerization of prolines, interconverting this amino acid between *cis* and *trans* isomers [33]. There are at least 60 reported cyclophilins found in plants, fungi, animals, and bacteria. Among them, 17 isoforms are found in humans and all of them are highly structurally conserved in the PPIase domain [34,35] (Figure 2). Human Cyp isoforms have shared PPIase activity, but differ in their subcellular localization. For example, CypA, CypB, and CypD localize to the cytoplasm, endoplasmic reticulum (ER), and mitochondria, respectively (reviewed in [36]). Interestingly, some Cyps are secreted from cells and may function as intercellular mediators, although their extracellular roles remain poorly understood [37,38,39,40].

Cyps have been implicated in the replication of many viruses, including human immunodeficiency virus-1 (HIV-1) [41], HCV [32], flaviviruses [42,43,44,45], and coronaviruses [46,47,48,49,50,51,52], among others [53]. Accumulating evidence suggests that Cyps interact with viral proteins to regulate various aspects of viral replication, although the molecular mechanisms have remained unclear, precluding the development of effective Cyp-targeting antivirals. Nonetheless, Cyps are promising targets for broad-spectrum host-targeted antiviral therapy. Further studies are warranted to understand the roles of Cyps in viral infection and elucidate the specific antiviral mechanisms, which would enable the development of improved cyclophilin inhibitors as potent antiviral drugs. Over the past two decades, much effort has focused on unravelling the roles of Cyps in HCV infection. Here, we describe these research efforts and provide a comprehensive overview of the roles of Cyps in HCV infection, and in related flavivirus infections. Improved understanding of the requirement for Cyps during viral replication may pave the way for Cyp-targeted antiviral therapy to address current limitations of DAA therapy for HCV, as well as to provide new treatment perspectives for other currently untreatable viral infections.

## 2. Cyclophilins and HCV Infection

In 2003, Watashi et al. were the first to report that HCV replication was reduced in the presence of Cyp inhibitors, such as CsA [54]. Using genotype 1b replicon models, it was shown that CsA treatment inhibited viral RNA replication and decreased expression of viral proteins NS5A and NS5B, demonstrating the potential for CsA in HCV therapy [54,55]. Subsequently, CsA was shown to similarly inhibit replication of a genotype 2a replicon, albeit with slightly less potency [56]. Importantly, the anti-HCV activity of CsA was independent of its immunosuppressive function, as the calcineurin inhibitor FK506 did not affect HCV replication [54]. Due to the lack of an effective treatment for HCV at the time, CsA was added to the standard-of-care interferon (IFN) treatment regimen. By combining these two agents, patient outcomes were improved, with only mild side effects [57,58]. Since then, non-immunosuppressive CsA derivatives, such as NIM811, alisporivir, and SCY-635, have been developed and shown to potently inhibit HCV replication in cell culture models [27,28,29]. Furthermore, some non-immunosuppressive CypI have shown promising antiviral activity against HCV in clinical trials [59,60]. Interestingly, treatment with the non-immunosuppressive CypI SCY-635 not only suppressed HCV replication, but also restored innate immune responses in patients chronically infected with HCV [60].

The inhibitory activity of CsA and other CypI against HCV replication suggested a role for Cyps during HCV infection, which was supported by mechanistic studies. Using a subgenomic replicon model, Nakagawa et al. showed that silencing of CypA, CypB or CypC expression in Huh7 cells by RNAi suppressed HCV genotype 1b replication [61], although silencing of CypA expression had the most profound impact on viral replication. Conversely, Watashi et al. showed that silencing of CypB expression, but not CypA expression, inhibited HCV genotype 1b replicon replication in MH-14 cells (a derivative of Huh7 cells) [62]. In yet another study, Yang et al. stably silenced expression of CypA, CypB or CypC in Huh7.5 cells, and found that only CypA was essential for replication of both genotype 1a and genotype 1b replicons [63]. Interestingly, Yang et al. also used the HCVcc/JFH-1 (genotype 2a) system to show that silencing of CypA expression profoundly inhibited authentic HCV infection in Huh7.5 cells [63]. Subsequent studies sought to clarify the roles of Cyps in HCV replication and address the controversial findings in the literature. Chatterji et al. silenced expression of CypA, CypB, CypC or CypD in Huh7 cells, and evaluated the impact on HCV Con1 (genotype 1b) replicon replication. An inhibitory effect on HCV RNA replication and viral protein expression was observed only in cells lacking CypA [64]. Consistently, Kaul et al. showed that silencing of CypA expression, but not CypB expression, in Huh7-Lunet or Huh7.5 cells inhibited replication of genotype 2a HCV, evaluated using subgenomic replicon and assembly-competent full-length RNA models [65]. Importantly, the inhibitory effect of CypA silencing could be reversed upon re-introduction of CypA, confirming a specific role for CypA [64,65]. Furthermore, studies with CypA mutants showed that the peptidyl-prolyl isomerase activity of CypA is required for HCV replication [64,65]. These studies led to the conclusion that CypA is the key player in HCV infection.

To further understand the antiviral mechanism of CsA against HCV, Fernandes et al. took an unbiased approach and selected for resistance to CsA in HCV genotype 1b replicon cells. After mapping the mutations that decreased susceptibility to CsA, it was found that mutations present in NS5A and NS5B affected the susceptibility of HCV replication to CsA [66]. Notably, mutations in NS5A conferred CsA resistance without concomitant mutations in NS5B being required, while the NS5B mutations alone conferred only a slight change in susceptibility to CsA, suggesting that CsA acts by two separate mechanisms on NS5A and NS5B, or through a single antiviral effect on the NS5A/NS5B complex. In either case, these findings were indicative of an interaction between Cyps and non-structural proteins NS5A and NS5B. Indeed, the viral RNA polymerase NS5B was shown to interact with both CypA and CypB [64,66], and binding of CypB to NS5B was found to stimulate polymerase activity, with a loss of CypB-binding leading to a decrease in HCV RNA replication [62]. Interestingly, one study reported the selection of NS5B mutants that were resistant to CsA; these mutations in NS5B conferred an increased ability for NS5B to bind RNA in the presence of CsA. Complementary studies supported these observations by showing that CypB increases RNA synthesis through binding to NS5B [67], although in vitro enzyme assays suggested that CypB may activate NS5B in a genotype-specific manner [68]. These findings are consistent with a role for Cyps in regulating HCV RNA replication.

More recently, research efforts have evaluated interactions between CypA and NS5A, focusing on domain 2 (D2) and domain 3 (D3). Both NS5A-D2 and NS5A-D3 are intrinsically disordered, leading to the hypothesis that Cyp binding to these domains may induce protein conformational changes required for NS5A activity. NS5A-D2 is critical for viral RNA replication [69], while NS5A-D3 contributes to viral particle production and assembly [70]. Biochemical studies identified a direct interaction between NS5A-D2 and the isomerase active sites of CypA and CypB [71], and other studies similarly showed that NS5A-D2 interacts with the CypA isomerase pocket [72,73]. Proline residues in NS5A-D2 were identified as putative substrates for CypA PPIase activity [71,74,75]. Notably, CsA treatment selects for mutations in a proline-rich region of D2 that overlaps with the CypA-binding site. These mutations, especially the NS5A D316E/Y317N double mutant, confer resistance to CsA across multiple HCV genotypes [76] and were proposed to decrease the requirement of NS5A for Cyp-mediated isomerization [75], thus enabling NS5A to carry out its functions independently of CypA.

**Figure 3:**
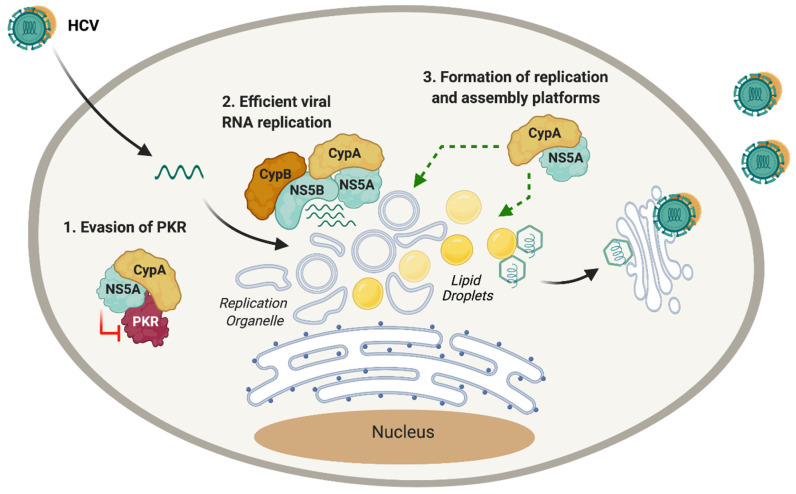
Suggested roles of cyclophilins during HCV infection. Cyps have been shown to have roles in several post-entry steps of HCV infection. (1) Formation of the CypA–NS5A complex inhibits the function of PKR, preventing activation of PKR-related antiviral immune responses. (2) The CypA–NS5A complex binds to the RdRp NS5B, enhancing replication of viral RNA. CypB also acts as a cofactor of NS5B, facilitating viral RNA replication. (3) CypA is required for formation of the HCV replication organelle and for optimal lipid droplet formation and lipid trafficking, supporting viral assembly and egress.

Although the mechanisms are not completely understood, the CypA–NS5A interaction likely contributes to viral RNA replication by several mechanisms (Figure 3). First, interaction with CypA affects the ability of NS5A to bind RNA [77]. The binding of CypA to a proline-rich region motif in NS5A-D2 enhances its RNA-binding properties [73]. This was corroborated by Dujardin et al. in a recent study where CypA was shown to allosterically modulate the disordered NS5A-D2, regulating viral RNA replication efficiency [78]. CypA and NS5B share overlapping binding sites in NS5A-D2 [79], and Ngure et al. showed that the RNA-binding region in NS5A-D2 coincides with the binding site of CypA and NS5B [80], suggesting the formation of a ternary complex between CypA, NS5A and NS5B that regulates viral RNA replication [81].

In addition to these direct roles in regulating the HCV RNA replication machinery, CypA appears to support other steps of the HCV replication cycle mediated by NS5A (Figure 3). For example, cyclosporine D (CsD) [82], CsA [32], non-immunosuppressive CsA derivatives [32,83] and structurally unrelated CypI [32] were shown to inhibit RO formation, but did not appear to affect the integrity of already established ROs [82]. Furthermore, silencing of CypA expression abrogated RO formation, which could be restored by re-expression of wild-type (but not catalytically inactive) CypA [83]. Thus, CypA contributes to RO formation, likely through its interactions with NS5A. A CsA-resistant HCV mutant (NS5A D316E/Y317N) was still able to form the double-membrane vesicles comprising the RO in the presence of CsD [82], which is consistent with a role for the CypA–NS5A interaction in RO formation. Thus, CypA appears to support formation of the HCV replication platform, which enhances RNA replication and may also contribute to evasion of innate immune responses, as the RO was shown to shield HCV RNA replication intermediates from innate immune recognition [16].

Interestingly, multiple studies have shown that CypI treatment more potently inhibits replication of full-length HCV compared to subgenomic replicon models [32,65,84]. While a possible link to polyprotein cleavage kinetics and the role of NS2 (which is lacking in subgenomic replicons) was explored [65,84], other studies identified a role for CypA in the HCV assembly process. Biochemically, CypA was shown to interact with NS5A-D3 [85], which regulates viral assembly [70], suggesting roles for Cyps in HCV assembly. An interesting observation was that CypI treatment of replicon or HCV-infected cells (but not uninfected cells) led to an increase in lipid droplet size and a decrease in lipid droplet number [86]. As lipid droplets are platforms for HCV assembly, it was hypothesized that the disruption to lipid droplets would inhibit assembly of infectious HCV virions. Furthermore, treatment with CypI, such as NIM811, impairs cellular lipid and protein trafficking, in the VLDL pathway [86], which HCV exploits during egress. These findings are consistent with the observation that CypI treatment decreases the release of infectious viral particles from HCV-infected cells [77,86].

CypA is also emerging as a regulator of PKR-dependent antiviral immunity during HCV infection. Daito et al. first reported that the expression of the IFN-stimulated gene (ISG) products was decreased in HCV-infected cells treated with IFN-α, compared to non-infected control cells [87]. However, treatment with CypI, such as SCY-635, restored the expression of ISGs at the protein level. A physical interaction between CypA and PKR was identified, suggesting that CypA regulates PKR activity [87]. Consistently, SCY-635 or CsA treatment of HCV-infected cells was found to reduce phosphorylation of PKR and downstream factor eIF2a, thus counteracting PKR-mediated shutdown of protein translation and restoring translation of ISGs [87]. Similarly, Gallay et al. reported that distinct CypI (including CsA, alisporivir, NIM811 and sanglifehrins), prevent the activation/phosphorylation of PKR in HCV-infected cells. It was proposed that activation of PKR through the accumulation of HCV dsRNA intermediates during viral replication reduces translation of ISGs, thus contributing to HCV evasion of the innate immune response [88]. Recently, Colpitts et al. showed that CsA and other CypI induce expression of IFN-β and ISGs at the mRNA level in a PKR-dependent manner [32]. It was proposed that CypA, under normal conditions, regulates the ability of PKR to mediate translation shutdown. In the presence of CypI, the interaction between CypA and PKR is disrupted, preventing PKR from shutting down translation (and thus restoring protein expression of ISGs), but enabling PKR to activate the transcription factor interferon-regulatory factor 1 (IRF1) [89], which induces the expression of antiviral genes with known anti-HCV activities [32]. Given that NS5A binds to and inhibits PKR [90], and that CypA binds NS5A [72,73], one possibility is that CypA regulates the ability of NS5A to inhibit PKR. Notably, binding sites for both CypA and PKR are located in D2 of NS5A, in the IFN-sensitivity-determining region (ISDR) that is associated with HCV sensitivity to IFN [90]. Although experimental validation of this model and the underlying mechanisms is still required, these studies collectively highlight an important role for CypA in HCV innate immune evasion. Consistently, Colpitts et al. showed that CsA treatment is more potent against HCV replication in innate immune-competent Huh7 cells compared to Huh7.5 cells [32], which have defects in innate immunity [91]. Similarly, CypA was required for HCV replicon replication in Huh7 cells, but not in Huh7.5 cells [32].

Despite the initial controversies surrounding the relative importance of CypA and CypB, and their roles in HCV replication, accumulating evidence suggests that both Cyps are necessary, although they likely have different roles in the replication cycle (Figure 3). This likely contributes to the strong antiviral potency of CypI against HCV infection, since by targeting multiple cyclophilins with distinct roles, CypI inhibit different aspects of the HCV replication cycle.

## 3. Cyclophilins and Other *Flaviviridae*

Flaviviruses, closely related to HCV, are also classified in the *Flaviviridae* family. Unlike HCV, however, flaviviruses are arthropod-borne viruses that cause a spectrum of disease, ranging from hepatitis to shock syndrome, encephalitis, and congenital abnormalities [92]. DENV is one of the most important arboviruses worldwide, mainly transmitted by female mosquitos belonging to the genus *Aedes* (*Aedes aegypti* and *Aedes albopictus*) [93]. It is estimated that there are 100 to 400 million DENV infections each year [94]. Flaviviruses can also emerge unexpectedly, such as the recent Zika virus (ZIKV) epidemic in the Americas [92] and the rapid spreading of West Nile virus (WNV) across North America since its first detection in New York in 1999 [95]. While there are vaccines for some flaviviruses, such as yellow fever virus (YFV) and Japanese encephalitis virus (JEV), the development of vaccines for DENV is challenged by the antibody-dependent enhancement effect (ADE), where live attenuated vaccines induce non-neutralizing cross-reactive antibodies that can enhance viral entry during a subsequent infection, particularly with a heterologous DENV serotype [96]. There are currently no approved antivirals for flaviviruses, leaving many people around the world vulnerable to endemic and emerging flavivirus infections.

Similarly to HCV, flaviviruses have (+)ssRNA genomes of ~11 kb in length that encode for a polyprotein that is processed into three structural proteins and seven non-structural proteins [97,98]. The flavivirus replication cycle begins with the attachment of the viral envelope glycoprotein (E) to cellular receptors, followed by internalization by clathrin-mediated endocytosis and subsequent low pH-mediated fusion [92]. Following the release of viral RNA into the cytoplasm, the (+)ssRNA is directly translated by cellular ribosomes into a polyprotein which is processed by NS3 and cofactor NS2B to produce structural and non-structural proteins [99]. RNA replication occurs within ROs derived from ER membranes through the activities of NS4A and NS4B [100], and requires the replicase complex comprised of RdRp NS5 and the NS3 helicase domain for RNA replication. New viral particles start to assemble as (+)ssRNA strands associate with capsid protein to form the nucleocapsid. Virions acquire their envelope through budding into the ER, and undergo a maturation process during egress, requiring processing of the Pr-M peptide into Pr and M by furin, resulting in the release of infectious viral particles [92].

Given the lack of antivirals for flaviviruses, and the promising antiviral effect of CypI against HCV, a related *Flaviviridae* family member, there has been interest in exploring CypI as flavivirus antivirals. In 2009, Qing et al. showed that silencing of CypA, CypB, CypC, or CypA and CypB expression in human hepatoma Huh7.5 cells reduces the replication of flaviviruses DENV-1, YFV, and WNV [42]. Viral replication could be rescued by adding back CypA with a functional PPI activity [42], supporting a specific role for CypA. Furthermore, CsA treatment was shown to inhibit DENV-1 [42], YFV [43], JEV [45] and ZIKV [44] replication. However, CsA still inhibited WNV replication in the absence of CypA, suggesting the involvement of additional Cyps [42]. Nevers et al. demonstrated that a small molecule CypI called SMCypI C31, which is structurally unrelated to CsA, exerts dose-dependent antiviral activity against multiple *Flaviviridae*, including HCV, DENV, YFV and ZIKV [101], opening perspectives for the development of non-immunosuppressive CypI as anti-flavivirus drugs. Some insights into the antiviral mechanisms of CypI against flavivirus replication are starting to emerge. Qing et al. showed that CypA interacts with WNV NS5 RdRp, suggesting a role for CypA in regulating viral RNA synthesis [42], similar to what has been described for HCV. Interestingly, proteomic studies revealed that CypA interacts with YFV NS4B [43], while another study showed that CypB binds to JEV NS4A [45]. Since both NS4A and NS4B have roles in RO formation [100], it is tempting to speculate that Cyps may contribute to flavivirus RO formation, as has been observed for HCV [32,82,83]. However, further studies are required to test this model and to understand the roles of Cyps in flavivirus replication. Furthermore, the roles of Cyps in the replication of other flaviviruses, including emerging viruses such as tick-borne encephalitis virus [102], Alkhurma hemorrhagic fever virus [103] and Powassan virus [104], have yet to be evaluated.

**Figure 4:**
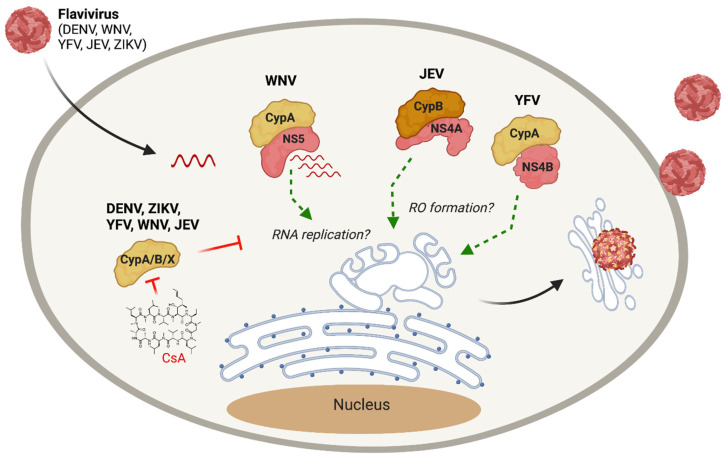
The role of cyclophilins in flavivirus infection. Treatment with CsA or other CypI inhibits DENV, YFV, WNV, ZIKV and JEV infection, although identification of the specific mechanisms and relevant Cyp(s) require further investigation. WNV NS5 RdRp interacts with CypA, which may contribute to viral RNA replication. Interactions between JEV NS4A and CypB, and YFV NS4B and CypA, may support viral replication by contributing to replication organelle formation.

Although much work remains to be done to clarify the mechanisms underlying the requirement for Cyps during flavivirus replication, potential roles of Cyps in flavivirus infection are beginning to emerge (Figure 4), providing a framework to guide future mechanistic studies and pave the way for host-targeting antiviral therapy for currently untreatable flavivirus infections.

## 4. Future Perspectives for Cyclophilin Inhibitors as Antiviral Therapy

**Figure 5:**
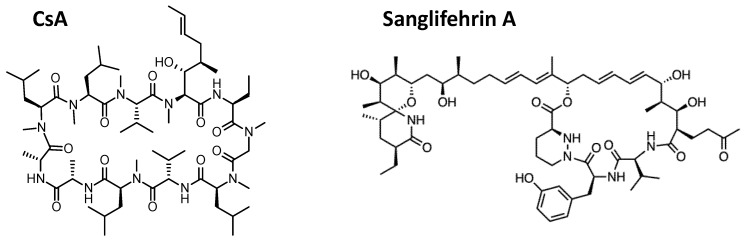
Chemical structures of CypI. CsA (**left**) and Sanglifehrin A (**right**) are shown.

Although its immunosuppressive activity has hampered the use of CsA as a clinical antiviral, CsA has been instrumental as a tool to understand the roles of Cyps in viral replication. Non-immunosuppressive CsA derivatives, such as alisporivir and NIM118, demonstrated strong efficacy as antiviral molecules in laboratory models of HCV infection and in clinical trials in HCV-infected patients, thus paving the way for the use of CypI in antiviral therapy. Recently, different types of CypI that are structurally distinct from CsA are being explored. Sanglifehrins are a group of cyclophilin-binding polyketides naturally produced by *Streptomyces* species [105]. Sanglifehrins are structurally distinct from CsA (Figure 5) and lack immunosuppressive properties. Interestingly, they have higher affinity for CypA and were shown to inhibit HCV genotype 1b subgenomic replicon replication more potently than CsA [105]. Consistent with the known CypI mechanisms against HCV replication, sanglifehrins disrupt formation of NS5A-CypA and NS5A-CypB complexes [105]. The sanglifehrin analog NV556 was found to inhibit HCV replication in vitro and in HCV-infected human liver chimeric mice [106]. Remarkably, a single 50 mg/kg dose of NV556 suppressed established HCV infection in these mice, with no viral rebound observed after 5 months [106]. Sanglifehrin derivatives with improved bioavailability have also been synthesized, and have demonstrated highly potent anti-HCV activity in the low nanomolar range in cell culture models [31,32].

To overcome limitations associated with CypI derived from CsA or sanglifehrin (such as poor cell permeability, potential off-target or immunosuppressive effects, and complex synthetic pathways), Ahmed–Belkacem et al. used a fragment-based drug discovery approach to identify non-peptidic, small-molecule cyclophilin inhibitors (SMCypI), unrelated to CsA or sanglifehrins [30]. SMCypI have potent PPIase-inhibitory activity, and were found to have potent antiviral activity against HCV genotype 1b replication [30]. The lead SMCypI, termed C31, was subsequently shown to disrupt the CypA–NS5A interaction [101], similar to what was shown previously for CsA. Further suggesting that SMCypI acts by the same mechanisms as CsA, C31 treatment selected for resistance mutations in NS5A-D2 [101] (genotype 1b D320E/Y321H; analogous to the D316E/Y317N mutation in genotype 2a selected by CsA treatment [76]). Importantly, C31 demonstrated antiviral activity against other *Flaviviridae*, including DENV, ZIKV and YFV, albeit with less potency than against HCV [101]. Nonetheless, these findings highlight the potential for developing CypI as broad-spectrum anti-*Flaviviridae* drugs; further structure-activity relationship studies aimed at improving the antiviral potency through chemical modification are warranted.

Proteolysis-targeting chimeras (PROTACs) harness the ubiquitin-proteasome system to induce degradation of target proteins. PROTACs are emerging as attractive candidates for anticancer therapies [107], and are being explored as antiviral drugs. Colpitts et al. recently synthesized a CsA-derived PROTAC molecule, modified with a ligand to recruit the von Hippel–Lindau E3 ligase to CsA targets [32]. This molecule, termed CsA-Prtc1, induced rapid proteasomal degradation of CypA and CypB, and potently inhibited HCV genotype 2a replication, with no immunosuppressive or cytotoxic effects [32]. Overall, these and other new approaches are paving the way for innovative antiviral strategies to bolster current efforts in the design and development of Cyp-targeting drugs.

## 5. Conclusions

Given the broad requirement for cyclophilins as viral host factors, CypI are attractive candidates for the development of host-targeting antivirals to treat multiple viral infections. Here, we reviewed the molecular mechanisms underlying the roles of Cyps in *Flaviviridae* infection. Although we have focused here on HCV and flavivirus infection, it is worth noting that CypI have been shown to inhibit coronavirus infection, including SARS-CoV-2 [52]. Therefore, development of CypI as an antiviral therapy may protect against a broad range of RNA virus infections, including future emerging flaviviruses or coronaviruses. Further studies are needed to understand the antiviral mechanisms and develop CypI capable of exerting potent antiviral activities without compromising immune responses. However, efforts in the HCV field over the past decades have contributed greatly to the understanding of the roles of Cyps in viral infection, and will certainly inform future work characterizing the roles of Cyps in flavivirus and coronavirus infections. This work paves the way for the development of CypI inhibitors as antiviral drugs, to open perspectives for new approaches to treat HCV and many other viral infections.

## Data Availability

No new data were created in this review. Crystal structures presented in this article are available in the Protein Data Bank (PDB, https://www.rcsb.org/) entry 4IPZ; accessed on 26 January 2021.
